# Usability Challenges for Health and Wellness Mobile Apps: Mixed-Methods Study Among mHealth Experts and Consumers

**DOI:** 10.2196/12160

**Published:** 2019-01-30

**Authors:** Mei Shan Liew, Jian Zhang, Jovis See, Yen Leng Ong

**Affiliations:** 1 Alliance Manchester Business School Manchester United Kingdom

**Keywords:** mobile applications, health and wellness mobile applications, mHealth apps, health and wellness mobile applications users, mHealth users, health and wellness mobile applications insiders, mHealth insiders

## Abstract

**Background:**

By 2019, there will be an estimated 4.68 billion mobile phone users globally. This increase comes with an unprecedented proliferation in mobile apps, a plug-and-play product positioned to improve lives in innumerable ways. Within this landscape, medical apps will see a 41% compounded annual growth rate between 2015 and 2020, but paradoxically, prevailing evidence indicates declining downloads of such apps and decreasing “stickiness” with the intended end users.

**Objective:**

As usability is a prerequisite for success of health and wellness mobile apps, this paper aims to provide insights and suggestions for improving usability experience of the mobile health (mHealth) app by exploring the degree of alignment between mHealth insiders and consumers.

**Methods:**

Usability-related major themes were selected from over 20 mHealth app development studies. The list of themes, grouped into 5 categories using the Nielsen usability model, was then used as a framework to identify and classify the responses from mHealth expert (insider) interviews. Responses from the qualitative phase were integrated into some questions for a quantitative consumer survey. Subsequently, categorical data from qualitative mHealth insider interviews and numerical data from a quantitative consumer survey were compared in order to identify common usability themes and areas of divergence.

**Results:**

Of the 5 usability attributes described in Nielsen model,  *Satisfaction* ranked as the top attribute for both mHealth insiders and consumers. *Satisfaction* refers to user likability, comfort, and pleasure. The consumer survey yielded 451 responses. Out of 9 mHealth insiders’ top concerns, 5 were similar to those of the consumers. On the other hand, consumers did not grade themes such as *Intuitiveness* as important, which was deemed vital by mHealth insiders. Other concerns of the consumers include in-app charges and advertisements.

**Conclusions:**

This study supports and contributes to the existing pool of mixed-research studies. Strengthening the connectivity between suppliers and users (through the designed research tool) will help increase uptake of mHealth apps. In a holistic manner, this will have a positive overall outcome for the mHealth app ecosystem.

## Introduction

### Background

In October 2016, for the first time, internet usage from mobile devices exceeded that from desktop or notebook computers [1]. By 2019, 2.7 billion people will be smartphone users [2], thanks to double-digit growth in China along with emerging markets of Southeast Asia. The convenience of mobile devices with app software capabilities supports the use of such apps as a powerful tool to transform the delivery of medical care and health care. A 2016 estimate puts the total number of medical health apps available over 250,000; however, the overall number of downloads of such apps appears to be decreasing [3].

### Assessment of mHealth Apps

It is uncertain whether the plethora of mobile heath (mHealth) apps is truly effective in improving health and wellness outcomes. Despite the attempts by several researchers to establish a systematic evaluation framework, evidence of their efficacy remains sparse [4-6]. Nevertheless, various forms of usability research models that may be broadly applicable have been proposed [7].

Obstacles to efficacy evaluation of mHealth apps tend to be related to the relative newness of the technology and the explosive pace of market growth in the past decade. In a study focused on the evaluation of mHealth measurement methodology, reliability, and validity of data, Kumar et al [8] concluded that the main challenges are the effects of variability on time-intensive data collection and the lack of a gold standard to assess convergent validity. In addition, the number of apps makes it challenging to set common standards for effectiveness. A review of 75 controlled trials of mobile technology-based health interventions found that most interventions were of low quality despite being conducted in high-income countries, especially when there was high variability in the types of measured outcomes [9]. For health care systems already burdened by suboptimal outcomes and excessive costs, premature clinical adoption of these mHealth technologies may detract from, rather than contribute to, what is needed for true overall health improvement.

### Usability of mHealth Apps

Broadly speaking, “usability” as a concept is about product quality and user experience. Its use as an assessment criteria has been widely adopted in the software inspection world since its emergence in 1990 [10] and is the predominant development delivery target for mobile apps today [11]. It has been shown that without considering usability, mobile apps are unable to retain users; tracking data has shown that users typically allocate less than 30 seconds to learn how to use the app before abandoning it for alternatives, or simply give up using mobile apps for this purpose altogether.

The scope of usability was defined early on within the International Organization for Standardization standards as “Extent to which a product can be used by specified users to achieve specified goals with effectiveness, efficiency and satisfaction in a specified context of use,” outlining *Effectiveness*, *Efficiency*, and *Satisfaction* as measurable attributes [12]. In contrast, the Nielsen model [10] is commonly used in the assessment of mHealth apps due to the suitability of its attributes for the assessment of software products; this model measures *Efficiency*, *Satisfaction*, *Learnability*, *Memorability*, and *Errors* as attributes. Zhang and Adipat [11] also highlighted issues specific to mobile devices, such as the mobile context, hardware limitations, and data-entry challenges as additional considerations not addressed with these models.

### Research Focus

Despite the increasing willingness of consumers to try mHealth apps [12], it is important to understand and appeal to the motivations of the users and decrease barriers to “digital adherence.” User experience research is thus a critical component to the success of mHealth apps. These can be in the form of focus groups and interviews to identify relevant themes [13-15]. Such tailored approaches provide valuable insights that facilitate extension of the target consumer base to nonconventional populations such as the elderly or patients from lower socioeconomic segments. Response analysis may also be performed within the context of relevant models such as the Health Information Technology Acceptance Model and the Mobile Application Rating Scale [16] to allow for full coverage of technological assessment.

Alternatively, a way to improve upon intrinsic limitations of such post-hoc analyses is to track users’ behavior in real time, allowing for instant feedback and improvements [17-19]. The main limitation of this method is potential selection bias in consumers, which is unsuitable for a product designed for wider audiences.

Although there are some mixed-research studies for mHealth apps and wearables [20-23], their focus is mostly at the app level for selected app products or target limited audiences. There are few studies at the systems level that attempt to understand the degree of alignment between the supply and demand with respect to the business environment.

Even a well-designed research approach is still a tedious process where the respondent population size is often a constraint. Consequently, answers might lie somewhere in an area that is not captured within the framework of the designed research approach. Compatibility of the research question to its relevant use of research tools has to be explored. Using the simple tool of 5W1H to illustrate, studies have thus far been dedicated to addressing questions such as “where are the gaps,” “who are involved,” “how can we improve,” and even “why are the gaps present.” We would like to contribute content to the area of “what,” specifically, what should be the focus area(s) of usability for mHealth app development.

The overall objective of this study was to help improve the ecosystem of digital health care by providing concrete directions for mHealth app companies with regard to consumers’ real needs. By examining the priorities and degree of alignment between mHealth insiders and consumers, critical hidden roadblocks are uncovered.

### Design and Setting

After reviewing various research approaches described by Creswell [24], a mixed-methods research design was selected to collect both categorical and numerical data. Qualitative data obtained from mHealth app insiders through face-to-face interviews were analyzed and integrated into the quantitative survey of the end consumers. Over the past two decades, mixed methods have become increasingly popular [25], and this study can be a valuable contribution to the literature.

The rationale of using the mixed-methods design was to explore the usability and key concerns highlighted by mHealth app insiders without prejudice. The consumer data will be used to validate the themes identified by mHealth app insiders and provide crucial insights that will have been otherwise neglected by the experts, aiming to better synchronize the developmental roadmap for mHealth apps and clarify the priorities in the consumers’ needs within the mHealth apps market. Three specific objectives addressed in this study are (1) identification of the major concerns of usability with regard to mHealth apps from mHealth insiders (eg, product developers), (2) evaluation of the relative importance of the experts’ listed concerns in the mind of consumers, and (3) identification of significant themes on usability from the consumers that may otherwise be neglected by the experts.

## Methods

### Development and Integration of Usability Themes

Under the International Organization for Standardization code 25010, two components specifically involve the user perspective, namely, functional suitability and usability. Functional suitability is applicable at the product level, where specific product-related questions are needed for execution. On the other hand, usability can be tested at the systems level, providing an opportunity for researchers to design high-level exploratory research. Hence, this research study employed the Nielsen usability model with the aim of performing a system-wide alignment analysis between the mHealth insider and consumer [10].

For this study, usability-related major themes were selected from over 20 mHealth app development studies. The final list comprised 22 unique usability themes (Table 1). The list was then used as a framework to identify and classify the responses from mHealth insider interviews during the coding analysis. A total of 19 usability themes mentioned by mHealth insiders in the qualitative data-collection phase matched the identified usability themes. No additional usability themes were captured from the qualitative phase. To meet the objectives of this study, all developed usability themes were integrated into the consumer’s quantitative survey questionnaire.

### Participants and Sampling

mHealth insiders were broadly identified from two main industries: Medical Healthcare and Information Technology (IT). Target candidates were experts with more than 10 years of work experience in the domain of health care or software app development for the Asia-Pacific market. A total of 19 experts were shortlisted for the qualitative interviews.

SoJump (WJX) was used for the quantitative survey platform [48]. In addition to the extensive survey features, SoJump offers access to China’s market, which is a critical interest for Asia Pacific. The minimum sample size was set at 384 participants in order to achieve 95% confidence level based on the ~4.5 billion population within the Asia Pacific region [49].

### Data Collection and Statistical Analysis

**Figure 1:**
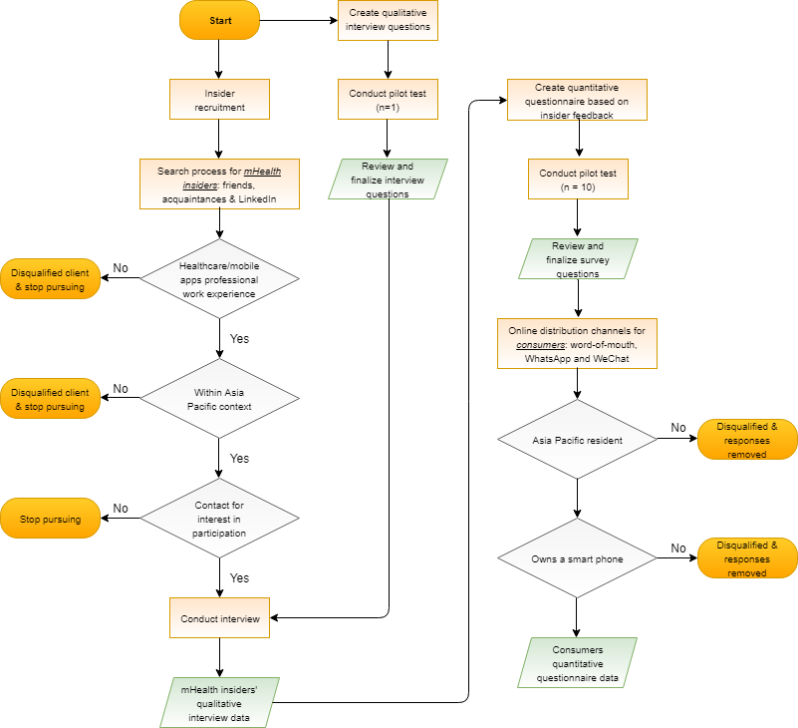
Qualitative and quantitative survey process map.

All the data for this study were collected within a 6-month period in 2017. Operationally, this mixed-research approach relied on lean design principles (Figure 1). The recruitment process for qualitative and quantitative components was based on selective and snowballing methods, respectively. As the qualitative interview fieldwork was more time consuming, qualified candidates were prescreened. Data-cleaning treatment was applied to assure data quality [50]. For example, for the quantitative online questionnaire, two eligibility questions were built into the survey and data from disqualified participants were removed after survey closure.

### Qualitative Study

Each interviewee in the panel of mHealth insiders signed a consent form that includes publication rights to the shared content. Written survey questions were provided to the interviewee during the process, which was also audio recorded. During each interview, all questions were sequentially asked, including the open-ended questions.

For the coding analysis of the transcripts, the authors adopted the Bengtsson method [51]. This 4-step data analysis process includes decontextualization, recontextualization, categorization, and compilation. To ensure scientific adequacy, both transcription of the audio file to text and coding analysis for each transcript were verified twice. A total of over 100 man-hours were spent in the qualitative interview data-collection phase. An industry expert assigned by the Alliance Manchester Business School, The University of Manchester, also validated the entire coding analysis processes and findings.

### Quantitative Study

**Figure 2:**
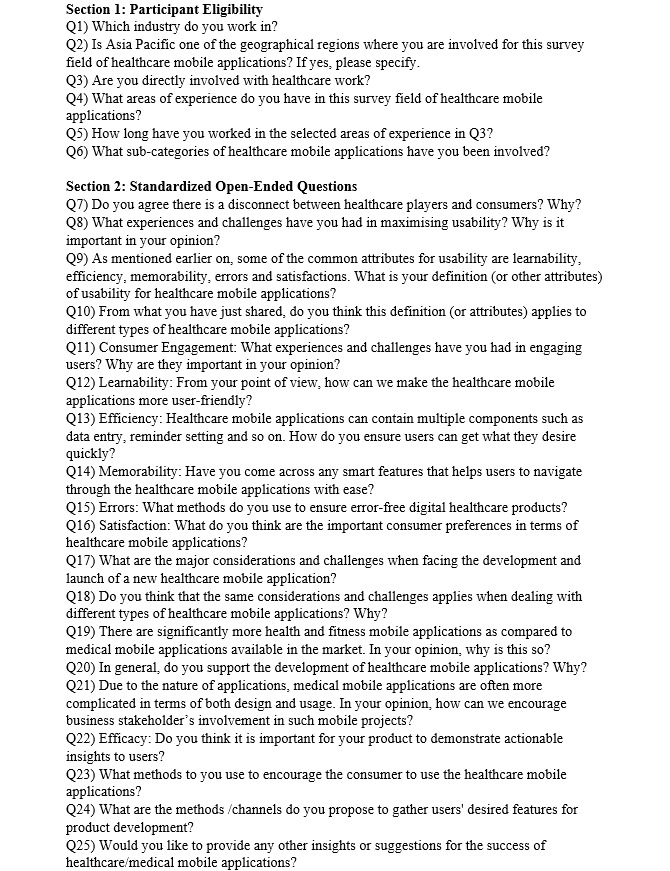
Qualitative study of the interview questions.

After coding analysis and theme identification from the qualitative study (Figure 2), relevant usability-related elements were incorporated into the consumer questionnaire. A pilot test with 10 consumers was performed for fine tuning.

The survey consisted of 34 questions [52] with a profiling section of 14 demographic questions, including qualifying questions to ensure that the respondents are from the Asia Pacific region and using smartphone devices. In addition, the survey platform captured participants’ location and device used, which helped in data processing. The questions were arranged in chronological order of consumers’ touch-point cycle, from initial awareness to engagement and postuse feedback. As this study focuses on mobile devices, the survey links were sent via mobile device-compatible messaging apps such as Whatsapp (Whatsapp Inc, Mountain View, CA), Line (Line Corporation, Tokyo, Japan), and Wechat (Tencent, Shenzhen, China). A total of 466 respondents completed the online survey.

The choice of input for usability perception questions follows the 5-point Likert scale, from least concern to most concern, or least important to most important. Likert data falls into the ordinal data type, and descriptive statistics include a mode or median for central tendency and frequencies for variability. To statistically claim that each concern is real for all samples, hypothesis testing for a proportion test with the frequency was conducted with a definition of null hypothesis and alternative hypothesis [53]. The one-sample Z-test was chosen for data analysis, as it is most compatible for ordinal data and the purpose is to test the population mean of frequency and determine the statistical significance.

Z-test follows the following formula [54]:

z_i_=(*P*_*i*_-*P*_*o*_)/((*P*_o_*[1-*P*_*o*_]/n)^0.5)

where *Pi* is the percentage of the population that shows concern or strong concern of 4 and 5 points, respectively, on the Likert scale.

**Table 1:** Usability themes. Some studies cover multiple themes. For simplicity, only the representative study is stated for each theme.

Usability category	Theme	Author, year	Study subject	Quantitative or qualitative
Learnability	Intuitive, users’ gestures are intuitive	Lin et al, 2017 [26]	Usability of data integration and visualization software for multidisciplinary pediatric intensive care	Quantitative
Satisfaction	Provide incentives	Rosario et al, 2012 [27]	A study in usability: Redesigning a health sciences library	Qualitative
Memorability	Familiarity, interface feels familiar and comfortable	Chevalier et al, 2014 [28]	The influence of the search complexity and familiarity with the website on the subjective appraisal of esthetics, mental effort, and usability	Qualitative
Memorability	Notification, utilizes useful notification alerts	Zeitz et al, 2016 [29]	Speed isn’t enough: Usability and adoption of an optimized alert notification system	Qualitative
Efficiency	Lean design, data allow seamless sharing across operating system devices	Bosse and Kelly, 2016 [30]	Improving EHR^a^ usability using lean methodology, studies in health technology and informatics	Qualitative
Efficiency	Efficiency, responsive and run smoothly	François et al, 2017 [31]	Digital, analogue, or redundant speedometers for truck driving: Impact on visual distraction, efficiency and usability	Qualitative
Efficiency	Actionable insights	Rose et al, 2017 [32]	Evaluating the usability of health insurance information with immigrant patients	Qualitative
Efficiency	IT^b^ compatibility, compatible with mobile device and required limited bandwidth	Juslstrom et al, 2011 [33]	Telecoil-mode hearing aid compatibility performance requirements for wireless and cordless handsets: Magnetic signal-to-noise	Quantitative
Efficiency	Responsiveness, regular updates in response to consumer needs	Green and Pearson, 2011 [34]	Integrating website usability with the electronic commerce acceptance model	Qualitative
Learnability	Integration ability - Technology, paired with latest technologies	Lee and Coughlin, 2015 [35]	An integrated approach to identifying determinants and barriers	Quantitative
Learnability	Integration ability - Lifestyle, usage integrated into daily life	Mishuris et al, 2016 [36]	Online diabetes-prevention program	Qualitative
Satisfaction	Enjoyable, fun and interesting to use	Putrino et al, 2017 [37]	Game-based therapy in stroke	Qualitative
Satisfaction	Functional deliverable, contributes to health objectives	Kawamoto et al, 2009 [38]	Enabling a semantically interoperable service-oriented architecture for healthcare	Quantitative
Satisfaction	Match expectation, understands targeted health concerns and key needs	Reed et al, 2016 [39]	Novel personal health technology to support early palliative care	Quantitative
Satisfaction	Addresses specific needs, consumer pain point	Stjernswärd and Hansson, 2017 [40]	Web-based mindfulness intervention for families living with mental health problems	Qualitative
Satisfaction	Active engagement, interactive and engaging	Jimison et al, 2008 [41]	Barriers and drivers of health information technology use for the elderly, chronically ill, and underserved	Qualitative
Errors	Health care experts’ involvement, advice from healthcare professionals	Kaipio et al, 2017 [42]	Usability problems do not heal by themselves: National survey on physicians' experiences with EHRs in Finland	Qualitative
Errors	Data accuracy	Ehrler et al, 2015 [43]	Usability of six data entry mobile interfaces for caregivers	Quantitative
Errors	Error free	Andreasen et al, 2017 [44]	Error-free text typing performance of an inductive intra-oral tongue computer interface	Quantitative
Satisfaction	Targets my demographic group	Armbrüster et al, 2007 [45]	The usability of track point and touchpad for middle-aged adults	Qualitative
Satisfaction	New features added frequently	Wolpin et al, 2015 [46]	Record title: Development and usability testing of a web-based cancer symptom and quality-of-life support intervention	Quantitative
Satisfaction	Progression analytics, providing visible progression on how much improvement, etc	Miah et al, 2017 [47]	Extending the framework for mobile health information systems research	Qualitative

^a^EHR: electronic health record.

^b^IT: information technology.

The *P* value is the probability of observing a sample statistic as extreme as the test statistic. Normal distribution cumulative probability was used to assess the probability associated with the computed Z-score. *P*_*o*_ is the hypothesized probability from the null hypothesis. The *P* value was then compared with the statistical confidence level, which was set as 95% [55]. The hypotheses that were accepted were considered statistically important themes and will be used as the basis for later discussion.

## Results

### Qualitative Interviews With mHealth Insiders

Qualitative interviews with mHealth insiders consisted mainly of open-ended questions to encourage them to express and elaborate their views. A total of 14 subjects were interviewed. This number falls within the proposed range of 5 to 25 participants for a phenomenological qualitative study [24,56]. One of the 14 candidates was disqualified due to insufficient experience in the Asia Pacific region.

**Figure 3:**
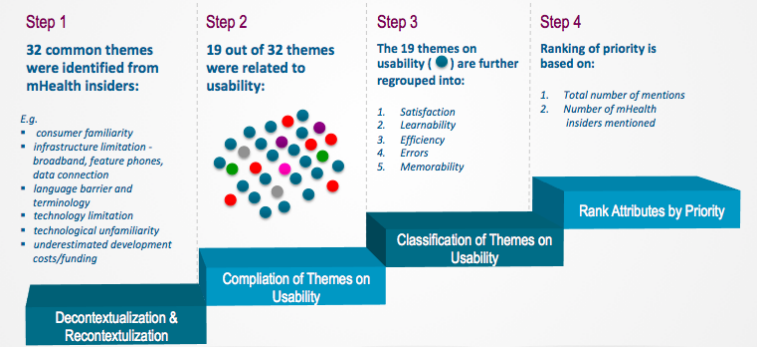
The 4-step data analysis flow for consumer interview.

The Bengtsson qualitative content-analysis method [51] was adopted and modified with a four-step analysis (Figure 3). The first step was to use decontextualization and recontextualization to identify all the common themes from coding analysis, followed by a typical content-analysis process, where the qualitative data were coded and the frequency of codes were counted and analyzed [57]. Next was the selection of themes only related to usability. Of the 32 common themes mentioned, 19 were usability related and the remaining 13 were mostly business and marketing related. The third step was to classify the usability themes into five categories based on the Nielsen definition [10], as described in Usability of mHealth Apps section. Finally, individual attribute priority was ranked based on the total number of mentions and the number of mHealth insiders who mentioned it.

**Figure 4:**
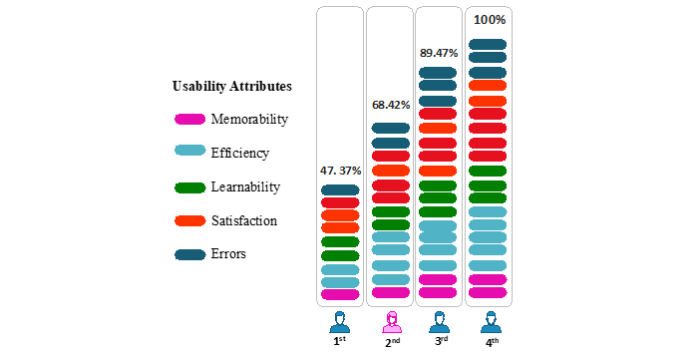
First observation to the 19 obtained usability themes.

**Figure 5:**
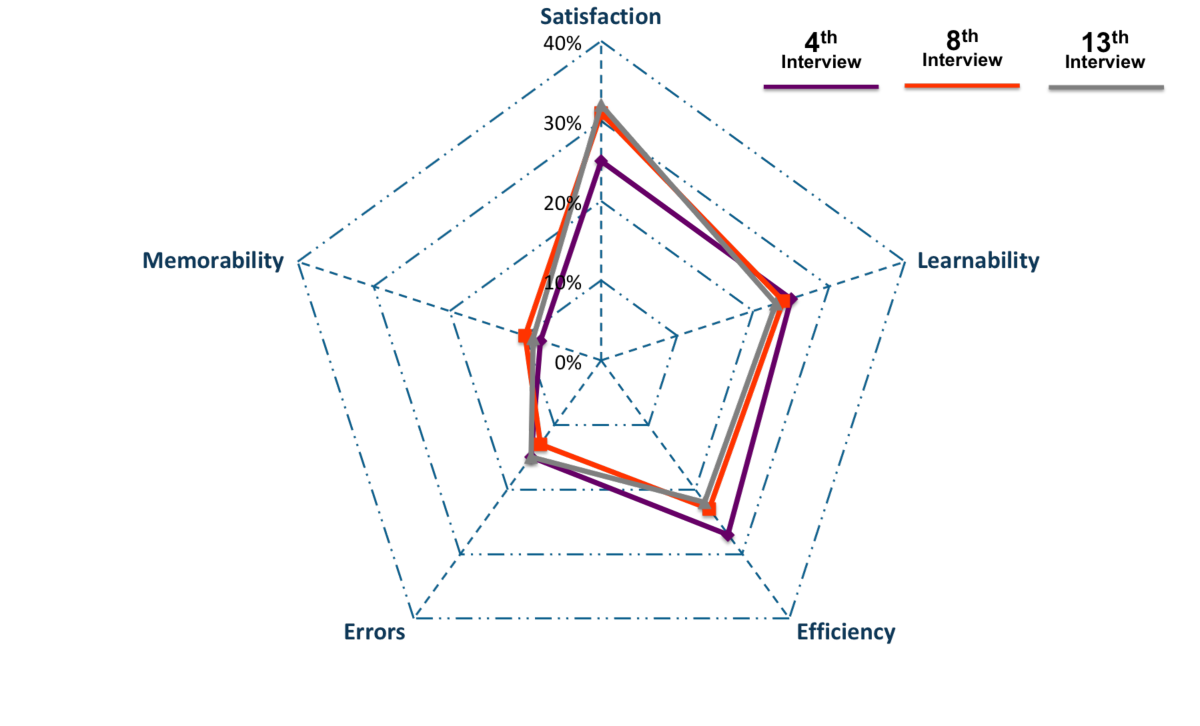
Second observation by frequency of mention.

Saturation testing was performed after completion of the qualitative study and content analysis. There is no common definition of saturation, but the generally agreed-upon principles and concepts are: no new themes, no new data or coding, and ability to replicate the study [58]. In this dataset, saturation for mHealth insiders’ interviews was tested based on two criteria. The first is at which point all 19 themes were mentioned; we observed that this was reached by the 4th interview (Figure 4). The second is based on frequency of mentions in each of the five usability categories, to observe when saturation of alignment of the proportion of mentions in the categories was reached. We found that the frequency of mentions after the 8th interview was aligned with overall response (Figure 5), with differences within 2% for each category. With the two abovementioned criteria met, we concluded that the number of interviewees was sufficient, and the data collected reached saturation.

### Emerging Themes of Focus for mHealth Apps

**Figure 6:**
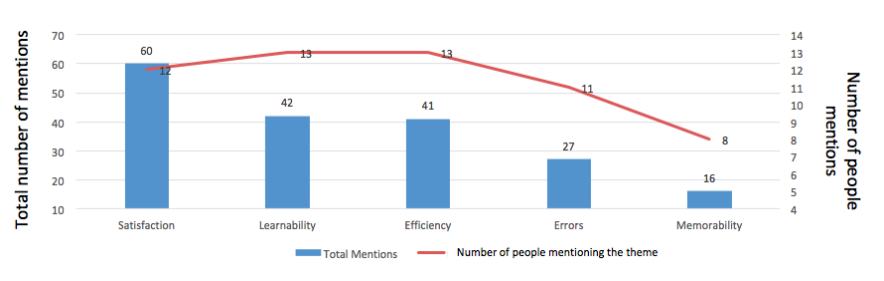
Usability overview from mHealth insiders.

Frequency of mentions was interpreted as an indicator of how the mHealth insiders perceived the importance or worthiness of a theme. The number of interviewees who mentioned a particular theme was interpreted as an indicator of how it has become a common understanding. The 19 usability themes were then summarized into an overview categorized by attribute (Figure 6). *Satisfaction* ranked as the overall most important attribute based on the number of total mentions, with 12 interviewees mentioning themes in this area. Additionally, although the total mentions were fewer, all insiders also discussed themes related to *Learnability* and *Efficiency*. Notably, questions were designed to avoid bias in favor of any particular attribute, which is why there is an unequal number of themes between each of the five usability attributes.

**Figure 7:**
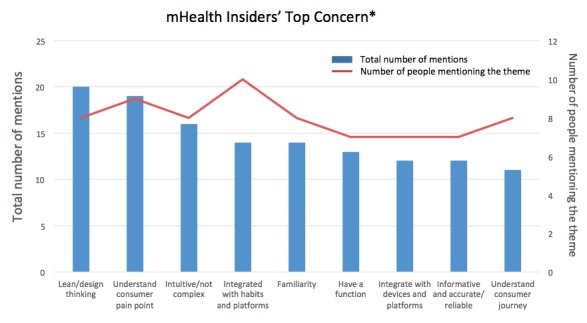
mHealth insiders' top concerns. Asterisk for top concern indicates total mentions≥10 (average) and number of people mentions≥7 (average). mHealth: mobile health.

**Figure 8:**
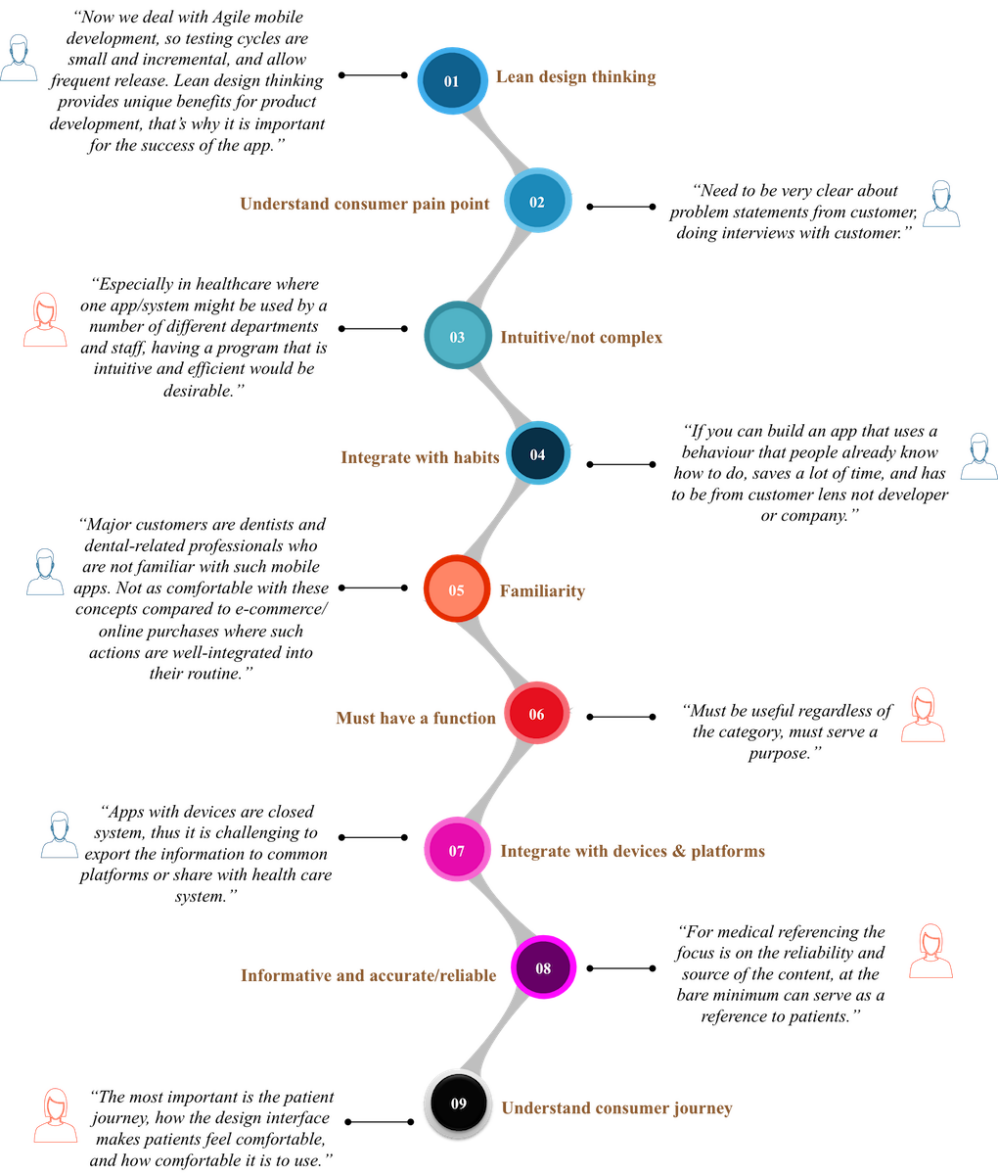
mHealth insider quotes for top concerns.

In the fourth step of data analysis, the 19 themes were prioritized based on the total number of mentions and the number of interviewees who mentioned them. Two synthetic conditions were then defined. First, the total number of mentions for that particular theme should be equal to or greater than the average number of mentions from all interviewees (≥10). Second, the number of insiders who mentioned the theme was equal to or greater than half of all respondents (≥7). With both conditions met, the themes were classified as “important.” A total of nine concerns met both criteria, with “lean design thinking” ranking as the top concern (Figure 7). Examples of quotes from the interviews that support the classification for the top concerns are also shown in Figure 8. The remaining 10 themes that did not meet the two criteria were classified as those of lower concern, with “notifications/getting attention” ranking the lowest in usability concern.

### Quantitative Survey Results of Consumers

A total of 466 responses were received in the quantitative survey phase, with 451 valid samples after data cleaning. Of these, 53% respondents were men, 97% were Apple and Android operating system users, and more than 90% had bachelor’s degrees or higher education. Respondents were from 11 Asian countries, with Singapore and China combined representing 90.9% of the total participants.

Three scenarios were evaluated: Threshold points set as 0.6, 0.7, and 0.8, where only 60%, 70%, or 80% of the population, respectively, agreed that it is a strong concern. All three scenarios passed the precondition check; thus, the Z-test could be used to determine whether the hypothesized population proportion differs significantly from the observed sample proportion [59].

**Figure 9:**
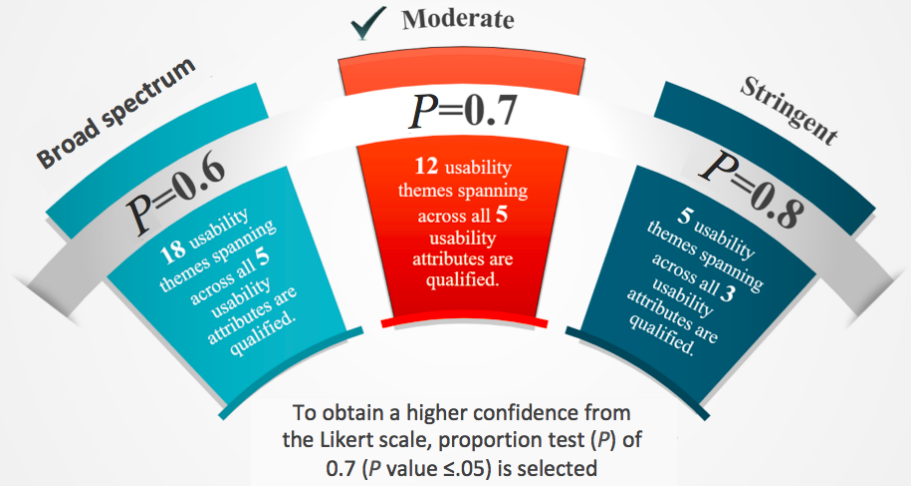
Sensitivity analysis for comparison of themes.

**Figure 10:**
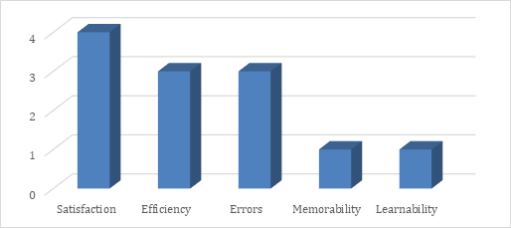
Number of top concerns from consumers.

As shown in Figure 9, when *P*_*o*_=0.6, 80% (18/22) of the themes across all five usability attributes were ranked important, which was insufficient to differentiate high versus relatively low concerns. Conversely, when *P*_*o*_=0.8, only 25% of the themes (5/22) from three attributes were considered important. Although the top priority is standing out, it may also eliminate concerns that are worthy of study. Under these constraints, a moderate *P*_*o*_=0.7 was selected to balance the opposing factors. Herein, the top concerns from mHealth insiders (12/22, with *P*_*o*_=0.7) were classified into their respective usability attributes (Figure 10).

**Figure 11:**
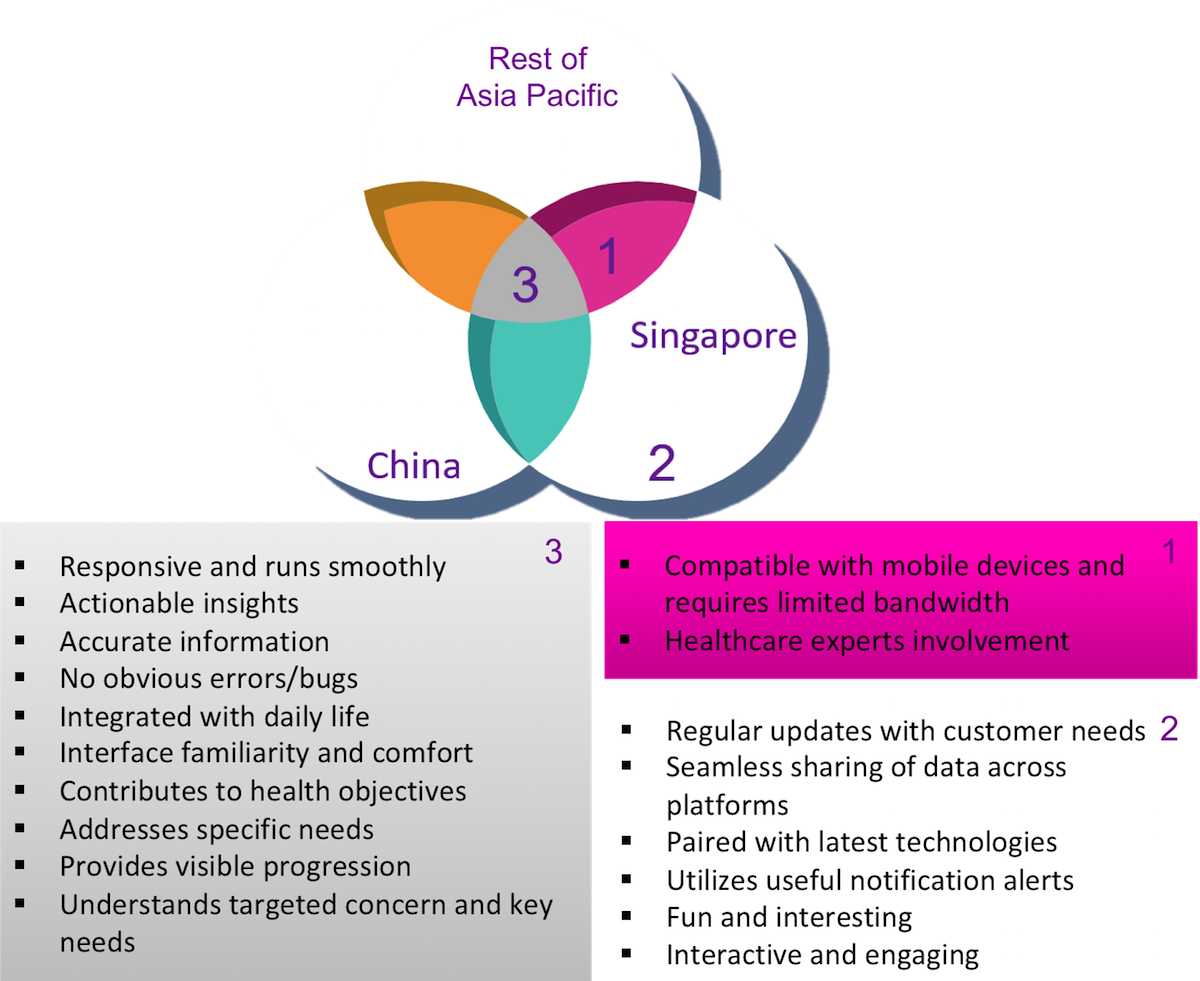
Demographic comparisons of mHealth insiders' top concerns.

Given the heavy skewing of respondents’ country, further analysis was performed to compare *overall response,* response from *China and Singapore combined,* response from *China only,* and response from *Singapore only*. To check if responses from respondents outside China and Singapore had any impact on the overall findings, the hypothesis test results from the *overall response* was compared against the response from *China and Singapore combined*. One item—*compatible with mobile device and requires limited bandwidth*—was not found to be of particular significance to the latter population. Diving deeper into this point, respondents from *China only* also did not consider this item as critical; however, it was considered critical for respondents from *Singapore only*. It is interesting to note that Singapore has the cheapest and fastest average bandwidth in Asia [60], suggesting that the lack of importance to China’s consumers may speak more towards the confidence in the quality of telecommunications access rather than just the cost consideration alone. The hypothesis test results of China versus Singapore versus the rest of Asia Pacific countries are summarized in Figure 11.

Ten themes common to China and Singapore and overall rated as high concerns and two themes*—compatible with mobile devices* and *healthcare experts’ involvement*—were high concerns in Singapore and overall. Furthermore, there were five top concerns specific to Singapore (Area #2 in Figure 11). One possible factor for these Singapore-specific concerns is that there were >25 respondents who were over 50 years old and 30 respondents with only high school and equivalent education as compared to only one respondent from China in each category. For these demographics, there is potentially a higher level of tension regarding digital technology in health care and a stronger need for more engaging apps. This could be confirmed with further studies in the future, with a sufficient sample size.

### Alignment of Priorities Between the mHealth Apps Insiders and Consumers

**Figure 12:**
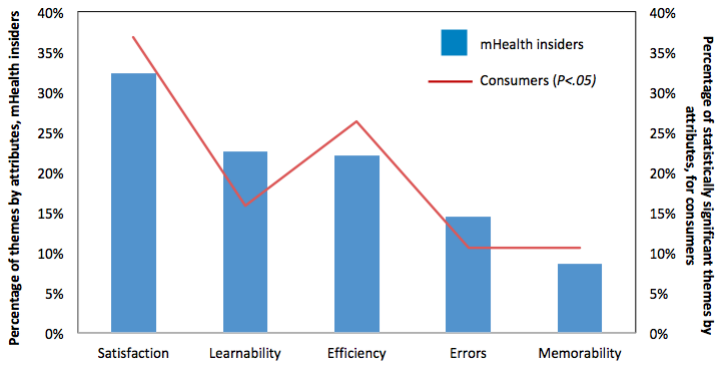
mHealth insider and consumer alignment study.

Methods were merged by transforming qualitative data into categorical counts and variables and then integrated with the quantitative database, called *integration with data transformation* [61]. The number of total mentions from insiders was normalized into percentages for easier comparison with the statistically significant themes selected by the consumers at the 95% confidence level. Figure 12 summarizes this comparison in a graphical manner.

There was a high degree of alignment between mHealth insiders and consumers in their top concerns. *Satisfaction*, *Learnability*, and *Efficiency* were the top three attributes for both groups, with *Satisfaction* ranking the highest and *Errors* and *Memorability* ranking lower. In terms of divergence, the degree of concern for *Satisfaction* was different, where stronger concern was observed from the consumers. For insiders, the concerns of *Learnability* and *Efficiency* were similar; however, consumers’ concerns of *Learnability* were less than those of *Efficiency*. One possible reason could be that once the *Efficiency* is met at a design level (eg, the three-click rule), there will be less reliance on *Learnability* for consumers. Hence, mHealth apps could be mastered with a gentler learning curve. The alignment of concerns in each theme is summarized in Table 2.

**Table 2:** Mobile health insiders’ top concerns versus consumer’s view.

Category	Subcategory	Mobile health insiders’ concern level	Consumers (α<0.05)	
			*P*_*o*_^a^=0.7	*P*_*o*_=0.6
Efficiency	Lean/design thinking	High	Reject	Accept
Satisfaction	Understand consumer pain point	High	Accept	Accept
Learnability	Intuitive/not complex	High	Reject	Reject
Learnability	Integrated with habits and platforms	High	Accept	Accept
Memorability	Familiarity	High	Accept	Accept
Satisfaction	Have a function	High	Accept	Accept
Learnability	Integrate with devices and platforms	High	Reject	Accept
Errors	Informative and accurate/reliable	High	Accept	Accept
Satisfaction	Understand consumer journey	High	Reject	Accept

^a^Hypothesized probability from the null hypothesis.

**Figure 13:**
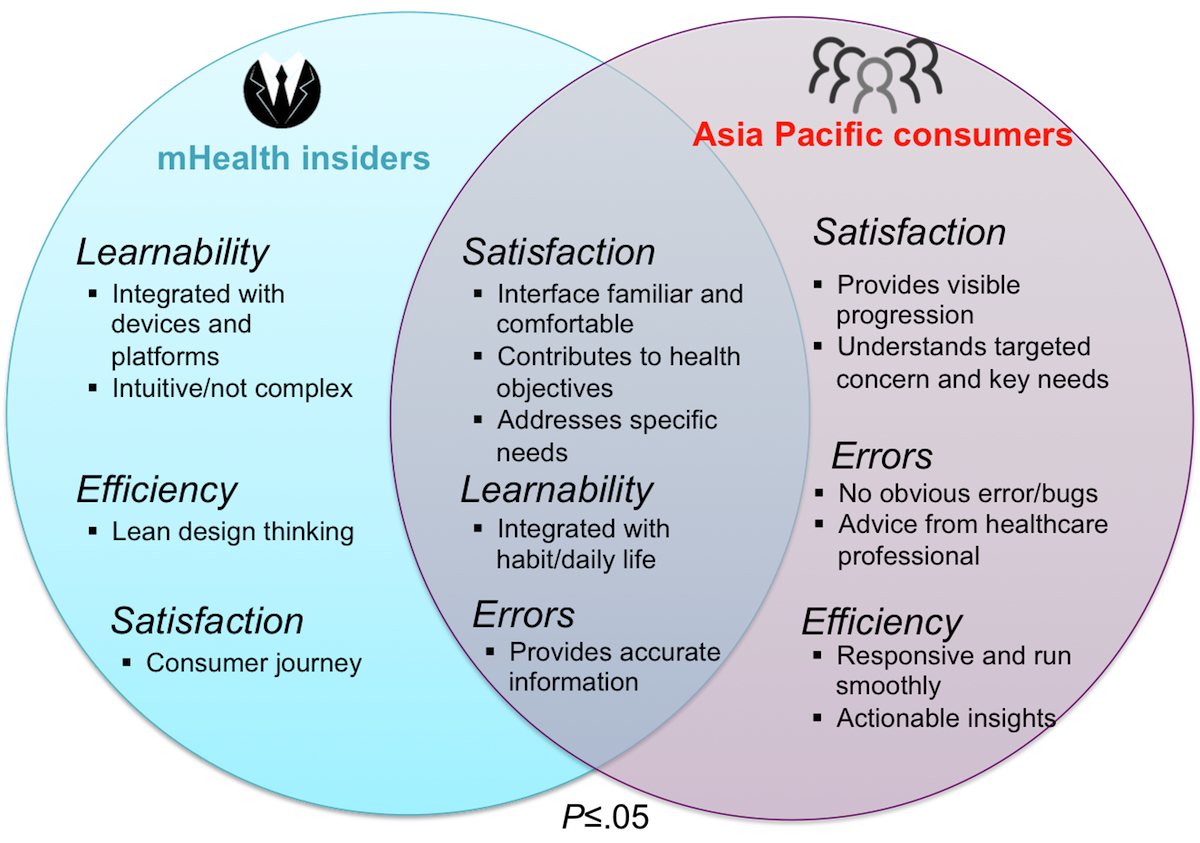
Comparison of top concerns from mHealth insiders versus Asia Pacific consumers.

Five of the nine mHealth insider’s top themes were also top rated for consumers (*P*<.05, *P*_*o*_=0.7), which also indicated a high level of alignment. *Intuitive functionality* was not statistically significant for consumers, possibly because these respondents already possessed a high level of technology awareness and comfort. Over 60% of the consumer population considered three themes— *lean design thinking*, *integrate with devices and platforms,* and *understand consumer journey* —a high priority (*P*<.05, *P*_*o*_=0.6). However, if we use 70% (*P*<.05, *P*_*o*_=0.7) as the gating threshold, these priorities are not as aligned. This suggests that they are still considered important, but not critical must-haves.

### Consumers’ Unique Perspective

Asia Pacific consumers were exposed to 22 known usability-related themes summarized from the literature, using a Likert scale rating. Of the 19 usability-related themes mentioned by mHealth insiders, 12 were statistically significant from the consumer’s point of view and 7 other themes were paid less attention. Interestingly, one particular theme—*to provide progression analytics* —highlighted by the mHealth consumers was not mentioned in the qualitative study.

The top consumer themes were whether the mHealth app contributes to their health objectives and whether it provides accurate information. This was verified by additional questions in the consumer journey section of the survey, where the top three reasons for them to stop using mHealth apps were: *not as useful as it claims*, *not user-friendly*, and *error or bugs affecting functionality*.

The overlap between the respondent populations is summarized in Figure 13. Commonly emphasized themes were mainly *Satisfaction* related. Some *Learnability*-related themes were ranked as top concern by mHealth insiders but not by the consumers, such as *integration with devices*, which may be a more technical consideration that consumers may already take for granted. In contrast, top concerns from consumers but not from the insiders were *Satisfaction*-related themes*,* followed by *Errors*- and *Efficiency*-related themes; several of these are indicative of various stages of the consumer journey, such as advice from health care professionals and provision of actionable insights.

### Other Important Themes Beyond Usability

Although unrelated to usability, the most-discussed theme from mHealth insiders by the total number of mentions is *the need to have a clear business model and plan for revenue stream*. This is nonetheless critical for the success of any mHealth app and serves as a major motivating factor driving app development in the first place. Thus, to test consumers’ willingness to pay for the use of such apps, a similar hypothesis study was done with different *P*_*o*_, *H*_*o*_ stating that consumers are willing to pay, and H_1_ stating that consumers are not willing to pay. The results indicated that fewer than 20% of consumers were willing to pay for both lifestyle/fitness apps and fewer than 35% were willing to pay for medical apps (Table 3).

**Table 3:** Population proportion test for willingness to pay.

Willingness to pay for a mobile app	*P*_*o*_^a^=0.4	*P*_*o*_=0.35	*P*_*o*_=0.25	*P*_*o*_=0.20
Lifestyle/fitness	Reject	Reject	Reject	Accept H_o_^b^
Medical	Reject	Accept H_o_	Accept H_o_	Accept H_o_

^a^*P*_*o*_: Hypothesized probability from the null hypothesis.

^*b*^H_o_: Null hypothesis

The finding suggests that although there is a need for revenue stream, it may not be a good choice to charge for the app itself or through in-app advertising. mHealth business owners will need to consider other options, for example, commission from institutions by introducing clients or developing monetization strategies using the consumer data collected.

Finally, other important themes rated important by the mHealth consumers included *adherence to governmental regulations* and *privacy protection*. To illustrate further, with *P*_*o*_=0.8, the r*espect my privacy* theme was significant, which suggests that privacy is a critical concern for more than 80% of the population. However, this theme was not highly mentioned by mHealth insiders, suggesting that developers may have underestimated the importance and impact of customer data protection and privacy. Although personal data protection legal enforcement is already an integral part of governmental regulations, consumer awareness and education of the existence and contents of such legal protections need to be enhanced. This can be achieved by putting efforts into marketing or enriching the in-app infographics for better illustration.

## Discussion

### Principal Results and Limitations

In this study, we utilized a mixed-research approach to show that mHealth insiders and consumers are aligned in assigning high priority to a number of usability aspects, particularly with regard to *Satisfaction*, *Learnability*, and *Efficiency* of mHealth apps, although they diverged in terms of the degree of concern for the attributes. The consumer quantitative study also highlighted unique perspectives that were not mentioned by the insiders. Although mHealth is merely the context for the study, we believe that, in general, early involvement and alignment of product development using customized mixed-research and synchronization between categorical and numerical data can result in specific, actionable insights.

The data presented in this paper are representative of a single point-in-time measurement. We speculate that longitudinal research spanning multiple time periods, coupled with real-life mHealth app usage and interaction, would provide deeper insights into individual user perspectives, especially within the fast-evolving digital environment.

The lack of access to a broad insider demographic may have also narrowed the scope of conclusions. Of the 13 mHealth insiders, only two had past experience straddling both health care and IT/app development. Despite a semistructured interview strategy, insiders were found to broadly discuss other topics (eg, sharing personal insights) that were out of the scope of study or provided a subjective view. However, the attributes identified were fairly aligned, and the study outcome was not too negatively impacted.

Separately, although the survey was released on an online platform accessible by the entire Asia Pacific region, we relied largely on personal connections for raising awareness and promotion. As such, respondents from Singapore and China constituted 88% of the total number of submissions, and conclusions made in the study are broadly applicable to these specific markets. Harzing [62] outlines several challenges of primary data collection facing researchers who pose cross-cultural empirical research, including language and translation barriers, cultural barriers, geographical distance, and the liability of foreignness. We anticipated and attempted to address some of these pitfalls, including the use of pilot testing in each country, translations by native speakers, and employing rankings where possible. Nevertheless, the bias of results is a factor that cannot be ignored.

The age range of survey participants was mostly between 31 and 40 years, with a majority of respondents holding a bachelor’s degree or higher education. This indicates a fairly young, well-educated population familiar with technology. The potential concerns here are twofold: the responses may not be captured for the specific case of mHealth apps that addressed chronic diseases of old age and the usability concerns of these digital natives may not be coherent with digital immigrants where the learnability bar may be set higher. For example, digital natives are already familiar with a new set of terminology, learning through interactive experience, and concepts of gamification [63], which are widely utilized tools in mobile apps. With a similar argument, only 22% of respondents indicated past usage of medical apps; therefore, survey responses overall may be viewed through experience with lifestyle wellness or fitness mobile apps only.

### Comparison With Prior Work

Compared with recent publications, there was general agreement on several aspects. Liu et al (2011) observed that mHealth apps that offered tracking, data visualization, or integration with Internet of Things devices are generally rated higher by users, despite these subcategories being in the minority of the apps on offer [64]. Similar to the findings of Cafazzo et al [65] and Boudreaux [66], consumers from this study were open minded about participating in app testing (35%) and giving feedback (42%) as part of the iterative improvement process.

Studies have also attempted to address potential differences in the level of acceptance correlated with socioeconomic status and health or digital literacy [67-70]. This is one aspect that we were unable to adequately address, as the reach of the survey did not encompass a broad spectrum of educational and socioeconomic levels or familiarity with technology (reflected in mobile usage, number of apps installed, etc). On the other hand, a qualitative study [14] showed that both digital natives and digital immigrants had comparable awareness and acceptance of mHealth apps and did not differ in their positive impressions of the value of apps.

Surprisingly, the most obvious divergence in our findings with the literature is in the relative importance placed by consumers on the opinions of health care providers during the decision phase of their consumer journey. Conventionally, medical professionals and formal bodies (eg, governmental agencies) have a strong influence on consumer choice [13], along with the impact of strategic initiatives put in place by national and global health organizations [66,71]. However, our study indicated that consumers still rely heavily on word-of-mouth recommendations from familiar sources that are typically not health care experts (65%). This difference indicates that use of mHealth apps is a consequence of subjective decision making rather than a balanced consideration of objective pros and cons.

### Application Relevance and Implication of Our Findings

There are two categories of applications in the direction of data maximization (extrapolating insights from current data into other applications) and use of the learning from this mixed-methods approach to address overall business initiatives (ie, supporting sustainable business and market research).

One attractive feature of sequential data collection is that it can also be interpreted separately. Potential new market segments can be identified from the consumer data. For example, there is a slight but noticeable shift in the *comfortability with using mHealth Apps*: Singapore’s consumers (12.4%) rated this attribute higher than China’s consumers (10.1%). Additionally, Singapore’s consumers (17.1%) were far more willing to respond to incentives to download apps than consumers in China (7.1%). For targeting China, which is driving the growth of mobile app usage in Asia, capturing these differentiating characteristics can be essential for success.

The mHealth insiders’ dataset can also be dissected to understand the viewpoint differences between experts from different backgrounds. This aspect is particularly significant in this study, as the expertise overlap between health care and software development is small and the impact of various misalignments on the business performance could be further investigated. As discussed, the assumption of a single point of perspective where experts from IT and health care are operating with parallel thoughts needs to be reformed.

The second potential application of this research work is in support of other business initiatives. Business sustainability is one such concern. mHealth apps are currently positioned as a value-added product or companion software tool for connected devices [72]. However, mobile phones today contain processors, sensors, and cameras powerful enough to collect and compute various physiological measurements. This implies that mHealth companies can make use of such alignment studies to develop and reposition mHealth apps as a stand-alone product. This reduces the reliance on electronic connected devices (eg, wristbands and imaging), which is also one of the biggest generators of excessive environmental waste, eliminating the need for the consumers to purchase companion devices and reducing their healthcare financial burden. Furthermore, this lowers the social stress of mastering both the hardware and software apps where there is no obvious value creation.

Another initiative is to maximize the dataset in the direction of generating a predictive modelling tool. Although relatively sparse, the existing dataset can serve as a framework to guide the design of additional studies, or an expansion of the respondent population, to multiply the size of the database. Large datasets will possess sufficient statistical power for creation of various models of consumer behavior and preferences at the discovery stage and increase the probability of creating winning product concepts. Considering the cost of mobile app development (from US $10,000 to >$100,000) [73,74] and the increasing market size [75], a modelling tool can help manage the risk and mitigate the negative costs.

Overall, the research data from this mixed-research study has served to develop ideas, initiate new inquires, and expand the range of inquiry. From concrete to speculative benefits, any future work that can target these applications are strongly encouraged.

### Conclusions

The goal of this research was to suggest directions for improvement in the design of mHealth apps for long-term benefits including improved mHealth app download rates, “stickiness,” and value within the entire digital mHealth ecosystem. A specific opportunity identified from the literature review is to assist health care companies to better focus on usability aspects rated to be of top importance by mHealth insiders and consumers.

Although the mixed methods used in this study were a more comprehensive and rigorous approach for comparing key interests of mHealth insiders and consumers, there are some challenges in data integration. Hence, there is a need to examine the space between mobile app developers, subject-matter experts, and consumers with regard to the parameters and relative priorities. We have strived to distil usability themes from qualitative interviews into the quantitative survey and vice versa. Alternative interpretations were explored, such as culture; reference standards for importance ranking; and demographic factors such as age, education, and tech-savviness influencing the survey results.

The major findings from this study not only addressed the three outlined research objectives, but also proposed an avenue of structured methodology for researchers of mHealth app development. Taken together, to the best of our knowledge, this is one of the first mixed-research alignment studies that has concretely identified gaps in both the theme and priority of usability concerns between mHealth insiders and consumers.

To conclude, although there is already good alignment of mHealth insiders and consumers in usability, much more could be done to better understand the needs and motivations of consumers with differing concerns. Areas have been identified where mHealth developers could place greater emphasis in their product-development cycle. Although other factors such as health care regulation and sustainable business models were not considered in this research study, they are important for the digital health care ecosystem to meet the demands in the Asia Pacific region and beyond.

## Abbreviations

IT: information technology
mHealth: mobile health
